# A Necroptosis-Related lncRNA to Develop a Signature to Predict the Outcome, Immune Landscape, and Chemotherapeutic Responses in Bladder Urothelial Carcinoma

**DOI:** 10.3389/fonc.2022.928204

**Published:** 2022-06-24

**Authors:** Jian Hou, Zhenquan Lu, Runan Dong, Guoqing Wu, Haibo Nie, Guang Yang, Cheng Tang, Genyi Qu, Yong Xu

**Affiliations:** ^1^ Department of Urology, Zhuzhou Central Hospital, Zhuzhou, China; ^2^ Division of Urology, Department of Surgery, The University of Hongkong-ShenZhen Hospital, ShenZhen, China

**Keywords:** bladder urothelial carcinoma, necroptosis-related, lncRNA signature, outcomes, immune checkpoint, chemotherapeutic response

## Abstract

**Objective:**

Many studies have drawn their attention to the immunotherapy of bladder urothelial carcinoma in terms of immunologic mechanisms of human body. These include immunogenicity of the tumor cells and involvement of long non-coding RNA (lncRNA). We constructed a necroptosis-related long noncoding RNA (nrlncRNA) risk factor model to predict BLCA outcomes and calculate correlations with chemosensitivity and immune infiltration.

**Methods:**

Transcriptomic data from BLCA specimens were accessed from The Cancer Genome Atlas, and nrlncRNAs were identified by performing co-expression analysis. Univariate analysis was performed to identify differentially expressed nrlncRNA pairs. We constructed least absolute contraction and selector operation regression models and drew receiver operating characteristic curves for 1-, 3-, and 5-year survival rates. Akaike information criterion (AIC) values for survival over 1 year were determined as cutoff values in high- and low-risk subgroups. We reassessed the differences between subgroups in terms of survival, clinicopathological characteristics, chemotherapy efficacy, tumor-infiltrating immune cells, and markers of immunosuppression.

**Results:**

We identified a total of 260 necroptosis-related lncRNA pairs, of which we incorporated 13 into the prognostic model. Areas under the curve of 1-, 3-, and 5- year survival time were 0.763, 0.836, and 0.842, respectively. We confirmed the excellent predictive performance of the risk model. Based on AIC values, we confirmed that the high-risk group was susceptible to unfavorable outcomes. The risk scores correlated with survival were age, clinical stage, grade, and tumor node metastases. The risk model was an independent predictor and demonstrated higher predictive power. The risk model can also be utilized to determine immune cell infiltration status, expression levels of immune checkpoint genes, and the sensitivity to cisplatin, doxorubicin, and methotrexate.

**Conclusion:**

We constructed a novel necroptosis-related signature that predicts BLCA outcomes and performs satisfactorily in the immune landscape and chemotherapeutic responses.

## Introduction

Bladder urothelial carcinoma (BLCA) is the 9^th^ most prevalent malignancy worldwide (1, 2). More than 199,000 people died from the disease in 2018, and more than 549,000 new cases were confirmed in 2018 (3). The number of BLCA events has been increasing worldwide over the past two decades, and the incidence of BLCA has been increasing yearly due to population aging and environmental pollution (4, 5). Treatment options for BLCA include transurethral resection, radical cystectomy, radiotherapy, and chemotherapy; nevertheless, BLCA remains an aggressive neoplasm with a substantial incidence of recurrence, metastasis, rapid progression, and unfavorable outcomes (6–8). One study in the United States estimated 80,470 new diagnoses of BLCA cases and at least 1,767 deaths in 2019 (9). Once the tumor has progressed and metastasized, the combination of systemic chemotherapy and surgery tends to be ineffective (10, 11). At least 30% respond to immunotherapies and immune checkpoint inhibitors (ICIs) (12). There is a need for research to construct reliable prognostic biomarkers through molecular profiling to identify prognostic markers and therapeutic targets for BLCA to improve outcomes.

Necroptosis is programmed cell death triggered by MLKL, RIP1, and RIP3 (13, 14). Several lines of evidence suggest that necroptosis is involved in Parkinson’s disease, infectious diseases, cancer, and other diseases (14, 15). Researchers found that necroptosis is a critical factor influencing tumor metastasis and T cell death (16). Interestingly, necroptosis has been related to antitumor immunity (13). As a substitute mode of programmed cell death to control apoptosis resistance, necroptosis plays a role in antitumor immunity in cancer therapy (13). Studies showed that necroptosis is a crucial cellular response that regulates many tumors’ onset, progression, and metastasis (17). Studies also found that necroptosis can serve as a biomarker in some diseases, particularly cancer (18, 19). Necroptosis enhances cancer cell migration and invasion in pancreatic carcinoma *via* the production of CXCL5 (20). Nevertheless, the precise functions of necroptosis in BLCA and the molecular mechanisms remain undetermined.

Long non-coding RNAs (lncRNAs) are a family of RNAs with no capacity for protein-coding that are more than two hundred nucleotides in length (21). A body of evidence suggests that necroptosis-related lncRNAs (nrlncRNAs) influence tumor progression and metastasis by triggering immune system processes and immune responses (17, 22). Studies showed that nrlncRNAs are associated with outcomes of various tumors (18, 22, 23). Bioinformatics analysis based on The Cancer Genome Atlas (TCGA) revealed that lncRNAs are associated with the progression of BLCA *via* immune-related pathways (24).

Differentially expressed nrlncRNAs may serve as prognostic indicators and drug targets in BLCA. This study utilized gene expression profiles of high-throughput sequencing data from TCGA to identify lncRNAs targeting necroptosis-related genes and to develop necroptosis-related prognostic signatures for patients with BLCA.

## Materials and Methods

### Datasets and Preprocessing

We queried TCGA data portal (https://tcga-data.nci.nih.gov/tcga/) (level 3 data, FPKM value) to obtain RNA sequencing profiles of 414 BLCA and 19 normal bladder specimens. We set the workflow type to “HTSeq-FPKM” and the data type to “Gene Expression Quantification” in the dataset download. To conduct subsequent analysis, normalization of the expression profiles to transcripts kilobase million values was carried out, and all analyses were conducted using R (version 4.1.1). Gencode (version 26) GTF files were obtained through Ensembl (http://asia.ensembl.org) for annotation and differentiation of lncRNAs and mRNAs (25). Sex, age, clinical stage, and survival rates were obtained from TCGA for clinical data after removing specimens with insufficient clinical data or a survival duration of 0 days. Finally, we included 19 normal bladder specimens and 408 BLCA specimens. **Supplementary Table 1** displays the clinical characteristics.

### Identification of Necroptosis-Related lncRNAs

We accessed the Gene Set Enrichment Analysis site (http://www.gsea-msigdb.org/gsea/index.jsp) and obtained the necroptosis gene set M24779, which included eight necroptosis genes. We combined prior reports on necroptosis and obtained 67 necroptosis-related genes, details of which are displayed in **Supplementary Table 2**. We calculated Pearson correlations between necroptosis-related genes and identified lncRNAs. Correlation coefficients > 0.5 and p < 0.001 were used to identify lncRNAs linked to necroptosis.

### Identification of Differentially Expressed Necroptosis-Related lncRNAs

We acquired 67 nrlncRNAs and used the R language version 4.1.1 “limma” package to identify differentially expressed necroptosis-related lncRNAs (DEnrlncRNAs) between BLCA and normal bladder specimens. The screening conditions were |log fold-change| > 1.0 and p < 0.05 (26). The expression matrix of differentially expressed lncRNAs was visualized using the heatmap package.

### Paired DEnrlncRNAs

DEnrlncRNAs were identified using several pairing cycles, assuming that C was equal to the sum of lncRNA A and lncRNA B. A 0 or 1 matrix was then created. If lncRNA A expression level was greater than the level of lncRNA B, C was defined as 1; otherwise, C was defined as 0. The 0-or-1 matrix was then re-evaluated. The correlation between pairs and patient outcomes was not evaluated if the expression level of lncRNA pairs was 0 or 1 because no pair ranks could accurately anticipate patient survival outcomes. In cases where the number of lncRNA pairs with an expression level of 0 or 1 accounted for more than 20% of the total logarithm, it was considered a valid match; otherwise, re-pairing was required.

### Development of a Necroptosis-Related Prognostic Risk Model

According to BLCA data obtained from TCGA, univariate Cox proportional hazards regression analysis was utilized to identify lncRNA pairs associated with outcomes from necroptosis-related lncRNAs (p < 0.05). We then performed least absolute contraction and selector operation (LASSO) regression with 10-fold cross-validation and a p-value of 0.05 and ran 1,000 loops. For each cycle, 1000 random stimuli were set to prevent overfitting. We then selected the best-paired combination to obtain nrlncRNA pairs in constructing the Cox risk coefficient model. By creating Cox univariate and multivariate analysis models, the risk coefficient for every necroptosis-related lncRNA pair associated with the outcomes of patients with BLCA was determined, and the risk score for each tumor specimen was determined. The aggregated risk score for every BLCA sample was the sum of the expression levels of each necroptosis-related lncRNA pair in the sample multiplied by the risk factor. The formula is as follows: $\text{risk score}=\sum_{i=1}^{n}{\text{risk coefficient }}_{\text{i}}\times \text{nrlncRNA expressioni.}$ The *survminer* and *survival* tools in R software were used to visualize the findings of the Cox analysis.

### Assessing the Predictive Power of Prognostic Risk Model

The area under the curve (AUC) was evaluated to ascertain the predictive capacity of the risk model for determining patient outcomes, and receiver operating characteristic (ROC) curves were produced using the survivalROC module in the R software, which included ROCs at 1, 3, and 5 years. To compute the Akaike information criterion (AIC) value at every point on the 1-year ROC curve to obtain threshold values that optimize the aggregate of specificity and sensitivity in separating low-risk from high-risk individuals, we conducted a Kaplan-Meier analysis to identify disparities in survival between individuals in the high- and low-risk groups, which we demonstrated using survival curves to calculate this cutoff value.

### Prognostic Risk Model Validation

We performed the chi-square test to examine the correlation between the model and clinical and pathological features to assess the clinical significance of the constructed model. The Wilcoxon signed-rank test was performed for these clinicopathological variables to examine the difference in riskScore between groups. The analysis findings were displayed using box plots. We conducted univariate and multivariate Cox regression analyses between clinicopathological parameters and riskScore to determine whether the model could serve as an independent outcome predictor. To present the results, we generated forest plots. *Survival*, *Heatmap*, and *ggupbr* were the R packages we used.

### Analysis of Tumor-Infiltrating Immune Cells

We used CIBERSORT to determine the association between risk scores and immune cell signatures (http://cibersort.stanford.edu/) (27), TIMER (version 2.0; http://timer.cistrome.org/) (28), QUANTISEQ (http://icbi.at/quantiseq) (29), Microenvironmental Cell Population Counter (30), EPIC (http://epic.gfellerlab.org) (31) and XCELL (http://xCell.ucsf.edu/) (32) to determine immune infiltration status in patients with BLCA. The Wilcoxon signed-rank test was performed to determine the differences in the content of immune infiltrating cells between the high- and low-risk groups of the constructed model. The findings were expressed in bubble charts. The *ggplot2* tool in R software was used to visualize the data.

### Analysis Between Immune Checkpoints and Risk Models

To investigate the relationship between the expression of immune checkpoint-related genes (TIM-3, PDL1, LAG3 PD1, GAL9, TIGIT, PD1LG2, and CTLA4) and the model, we compared high- and low-risk subgroups and visualized them using the *ggstatsplot* package and violin plots.

### The Value of Risk Models in Clinical Management

The half-inhibitory concentration (IC_50_) of frequently used chemotherapeutic medicines for BLCA was determined in the dataset to assess the model’s utility in medical therapy. The Wilcoxon signed-rank test was performed to calculate differences in IC_50_ between high- and low-risk groups. The data were presented using the R software packages *ggplot2* and *pRRophetic (* 33).

### Statistical Analyses

This study used R software (version 4.1.1) for statistical analysis. Differences between the two subgroups were estimated using the Wilcoxon rank-sum test. All statistical tests were two-way when p < 0.05 indicated statistical significance.

## Results

### Identification of DEnrlncRNAs

**Figure 1:**
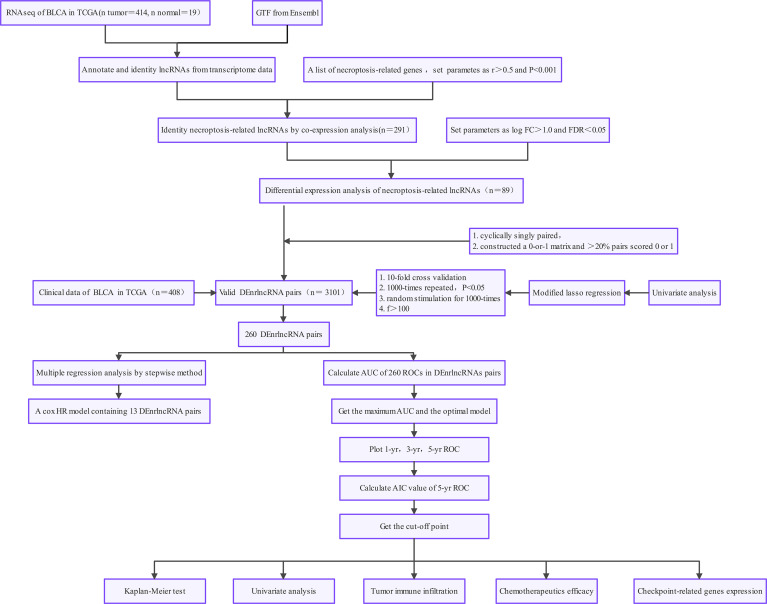
Study workflow.

**Figure 2:**
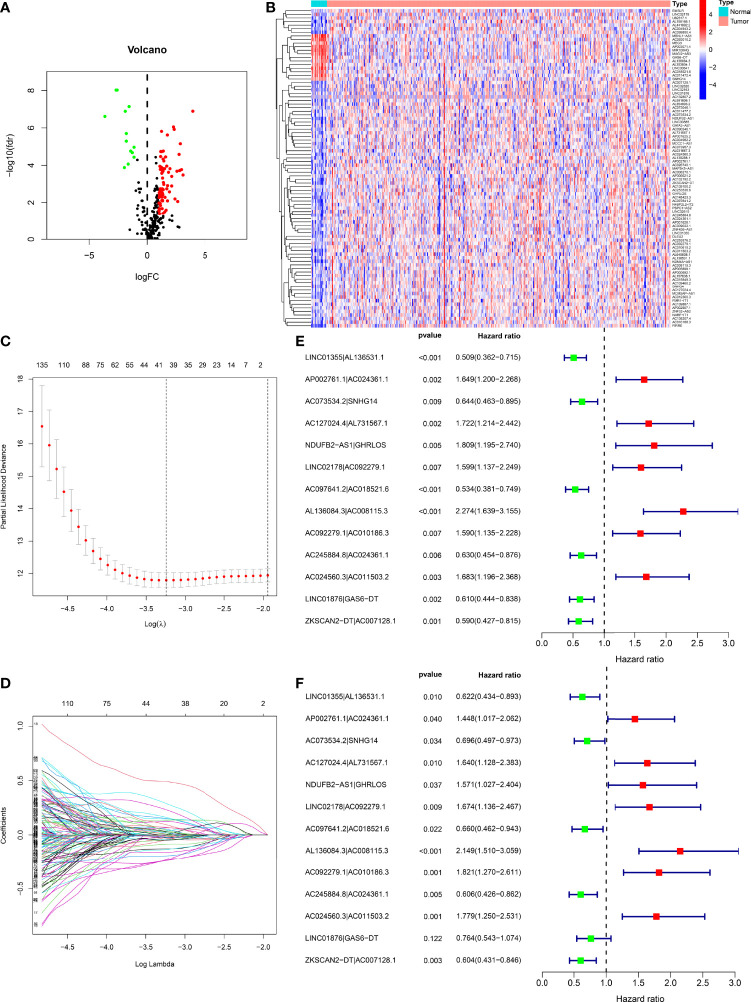
Development of a risk model with DEnrlncRNA pairs. **(A)** The volcano plot of necroptosis-related lncRNAs. **(B)** The heatmap of necroptosis-related lncRNAs between BLCA and normal tissues. **(C)** LASSO coefficient distribution of 13 necroptosis-related lncRNAs. **(D)** Ten-fold cross-validation for variable selection in LASSO models. **(E)** A forest map revealing 13 DEirlncRNA pairs detected using univariate Cox regression analyses. **(F)** A forest map revealing 13 DEirlncRNA pairs detected using multivariate Cox regression analyses.

**Figure 1** demonstrates this study’s flowchart. The initial step was to obtain transcriptome data for BLCA from TCGA. Finally, we included 19 normal samples and 414 BLCA samples. In the second step, data were annotated in accordance with the Gene Transfer Format files from Ensembl. We combined prior reports on necroptosis and obtained 67 necroptosis-related genes, according to 67 necroptosis-related genes and DEnrlncRNAs between normal and tumor samples (|log fold-change|>1.0 and p < 0.05), we identified a total of 291 nrlncRNAs (**Supplementary Table 3**), of which 89 were classified as DEnrlncRNAs (**Figure 2A**); 76 underwent upregulation, and 13 underwent downregulation (**Figure 2B** and **Supplementary Table 4**).

### Development of DEnrlncRNAs Pairs and a Risk Model

Using multiple rounds of matching of 89 DEnrlncRNAs, 3101 necroptosis-related lncRNA pairs were identified (**Supplementary Table 5**). Next, univariate Cox regression analysis was performed to extract 260 DEnrlncRNA pairs affecting survival (**Supplementary Table 6**). To create a risk model, LASSO regression analysis was conducted to identify 13 necroptosis-related lncRNA pairs (**Figures 2C, D**). Then, univariate and multivariate Cox regression analyses were conducted on these 13 nrlncRNA pairs (**Figures 2E, F**), and each nrlncRNA pair’s risk coefficient was calculated (**Table 1**).

**Table 1:** Thirteen pairs of prognostic necroptosis-related lncRNA pairs multivariate Cox regression analysis results.

LncRNAs	Coefficient	HR	HR.95L	HR.95H	P-value
LINC01355|AL136531.1	-0.4742	0.6224	0.4338	0.8929	0.0100
AP002761.1|AC024361.1	0.3702	1.4480	1.0168	2.0621	0.0401
AC073534.2|SNHG14	-0.3631	0.6955	0.4969	0.9735	0.0343
AC127024.4|AL731567.1	0.4944	1.6395	1.1278	2.3834	0.0096
NDUFB2-AS1|GHRLOS	0.4520	1.5714	1.0273	2.4039	0.0372
LINC02178|AC092279.1	0.5151	1.6738	1.1356	2.4670	0.0093
AC097641.2|AC018521.6	-0.4155	0.6600	0.4620	0.9428	0.0224
AL136084.3|AC008115.3	0.7651	2.1492	1.5099	3.0592	2.16E-05
AC092279.1|AC010186.3	0.5996	1.8214	1.2704	2.6113	0.0011
AC245884.8|AC024361.1	-0.5008	0.6060	0.4261	0.8620	0.0053
AC024560.3|AC011503.2	0.5758	1.7785	1.2497	2.5311	0.0014
LINC01876|GAS6-DT	-0.2690	0.7641	0.5434	1.0744	0.1218
ZKSCAN2-DT|AC007128.1	-0.5047	0.6037	0.4306	0.8463	0.0034

HR, hazard ratio; HR.95L, 95% CI lower limit; HR.95H, 95% CI upper limit.

### Evaluating the Risk Model’s Outcomes Predictive Capability

**Figure 3:**
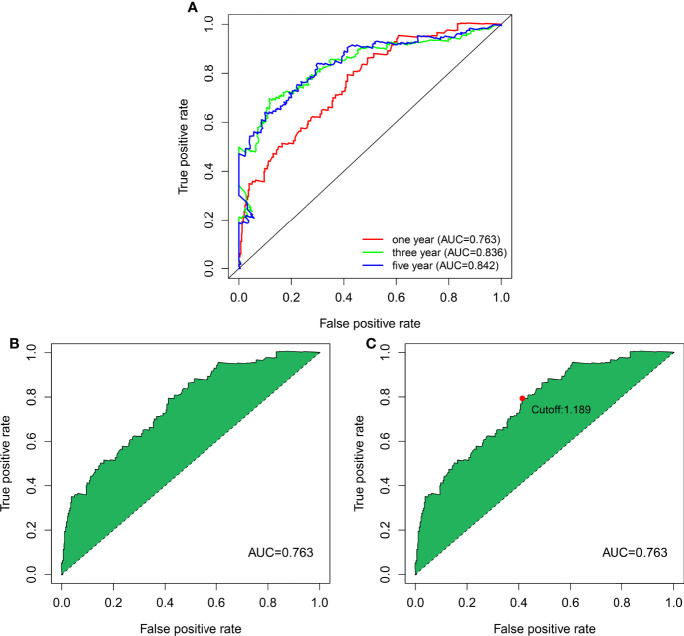
DEnrlncRNA pairs to establish a risk assessment model. **(A)** The 1-, 3-, and 5-year ROC curves derived by optimal model construction showed that all AUC values were above 0.763. **(B)** The 1-year ROC curve with the largest AUC value generated by the model. **(C)** The cutoff value of 1.198 for distinguishing high-risk and low-risk patients was generated using the optimal model.

The 13 nrlncRNA pairs were used to construct the 1-, 3-, and 5-year ROC curves of BLCA patients (**Figure 3A**), and the 1-year AUC was computed as a maximum of 0.763 (**Figure 3B**). The AUC values for three and five years were 0.836 and 0.842, respectively, demonstrating that this risk model can also be used to predict 3- and 5-year outcomes for BLCA. According to best fit, the threshold value for differentiating between high and low-risk groups of BLCA patients was 1.189 (**Figure 3C**).

### Clinical Assessment by the Risk Model

**Figure 4:**
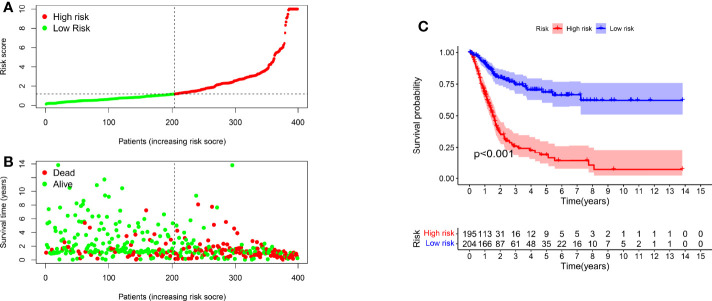
A risk model for outcome prediction. **(A)** Risk score for each patient. **(B)** Survival outcomes for every patient. **(C)** Kaplan-Meier curves based on the status of survival of patients in high- and low-risk groups.

**Figure 5:**
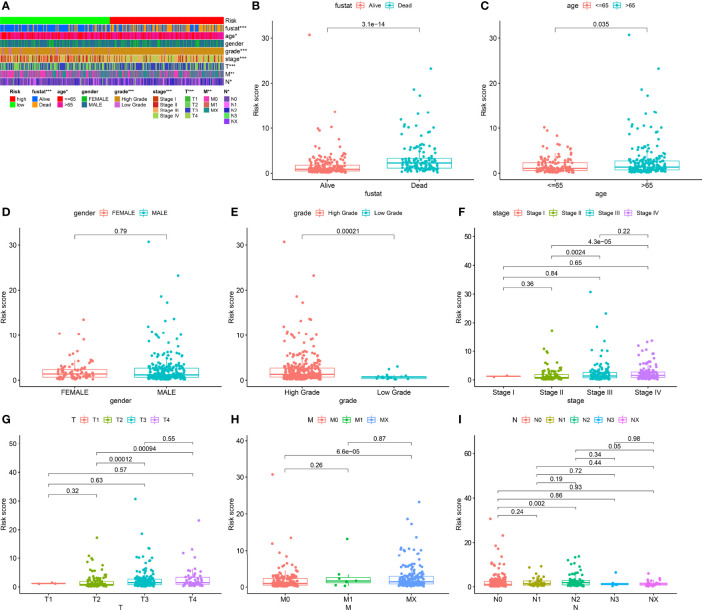
Clinical evaluation of risk models. **(A)** Heatmap of clinicopathological features. **(B)** Survival status. **(C)** Age. **(D)** Gender. **(E)** Grade. **(F)** Clinical stage. **(G)** T stage. **(H)** M stage. **(I)** N stage. *p < 0.05; **p < 0.01; ***p < 0.001.

**Figure 6:**
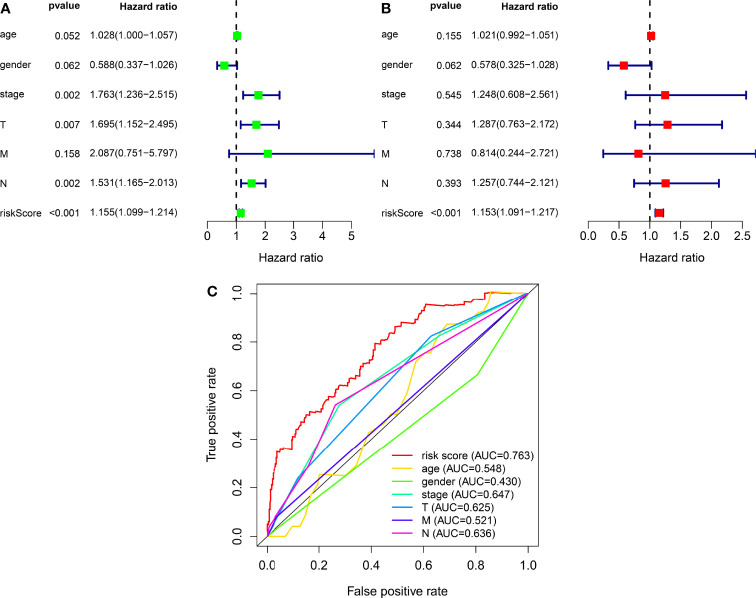
The independence of prognostic models and clinicopathological characteristics for BLCA outcome prediction. **(A)** A forest map of univariate Cox regression analysis of BLCA prognostic associations. **(B)** A forest map of multivariate Cox regression analysis of BLCA prognostic associations. **(C)** Comparison of AUC values for clinicopathological characteristics and risk scores.

We classified the patients into high- and low-risk groups based on the threshold value. We assigned 204 patients to the low-risk and 195 to the high-risk subgroup (**Figure 4A**). **Figure 4B** demonstrates the distribution of survival status in the subgroups. The high-risk subgroup had more deaths than the low-risk subgroup, and the differences in survival time between subgroups were compared. **Figure 4C** illustrates that, compared to high-risk patients, low-risk patients had better outcomes (p < 0.001). We then conducted several chi-square tests to determine the relationship between risk scores and clinical and pathological features and generated a heatmap using the Wilcoxon signed-rank test (**Figure 5A**). Scatterplots showed that survival (**Figure 5B**), age (**Figure 5C**), grade (**Figure 5E**), clinical stage (**Figure 5F**), T stage (**Figure 5G**), M stage (**Figure 5H**), and N stage (**Figure 5I**) were significantly associated with risk scores. Gender (**Figure 5D**) was not significantly associated with risk scores. Then, the univariate and multivariate Cox regression analyses were performed on risk scores and clinical correlation factors, and forest maps were drawn (**Figures 6A, B**). In univariate Cox analysis, we found that T stage, N stage, and riskScore were significantly associated with outcomes (**Figure 6A**). Multivariate Cox regression analysis revealed that only riskScore could be utilized as an independent predictor for BLCA (**Figure 6B**). ROCs were used to compare the differences in 1-year survival prediction performance. This risk model had the greatest AUC value (0.763) (**Figure 6C**), suggesting that it has an excellent capacity to predict outcomes.

### Correlation Analysis Between Risk Models and Tumor-Infiltrating Immune Cells

**Figure 7:**
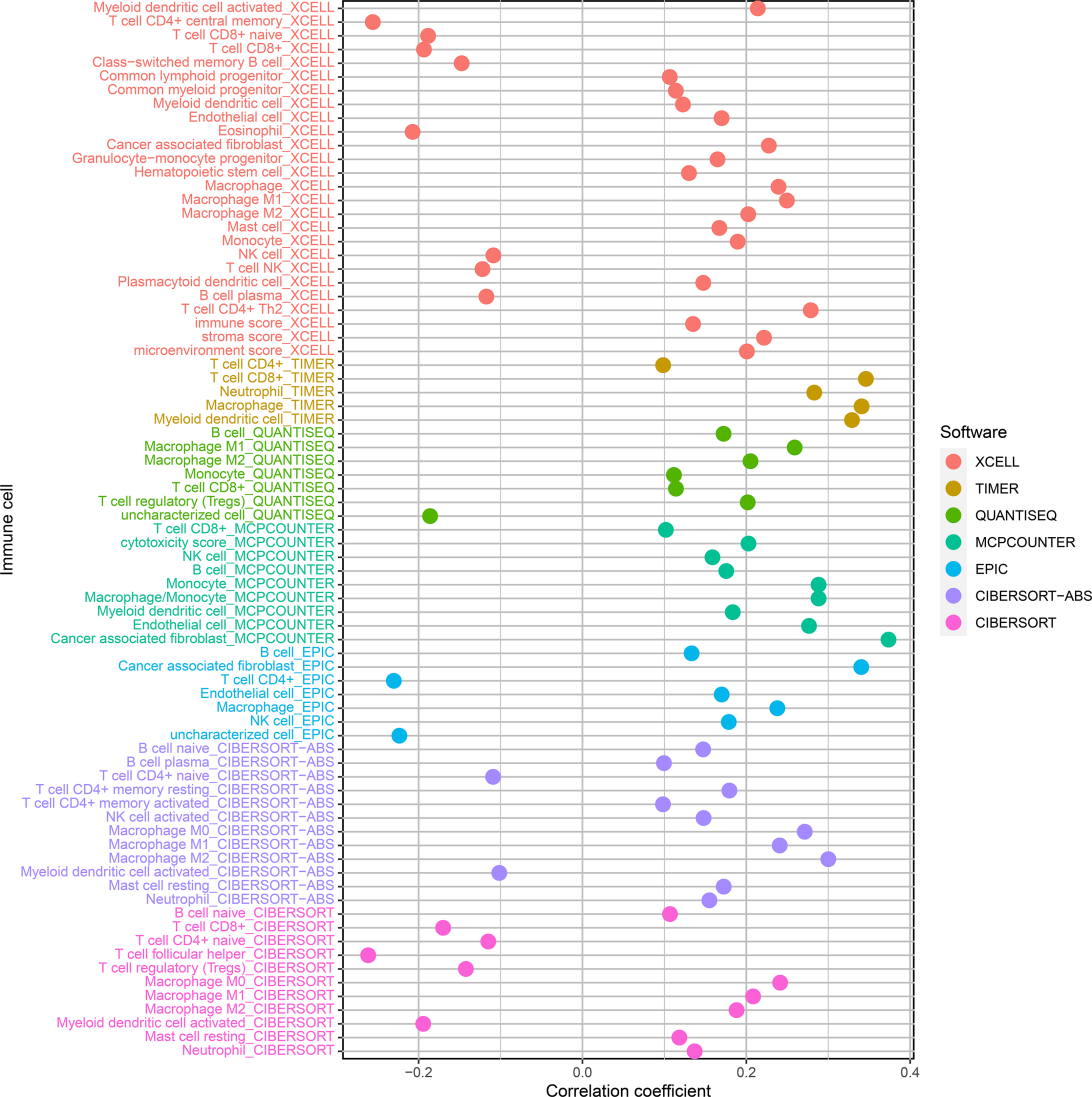
Correlation analysis of tumor-infiltrating immune cells and risk models of patients with BLCA.

We used CIBERSORT-ABS, QUANTISEQ, XCELL, EPIC, TIMER, CIBERSORT, and MCPCOUNTER, to study whether the risk model is correlated with the tumor immune microenvironment. The link between the risk model and tumor immune infiltrating cells was investigated by performing the Pearson correlation test. The screening criterion was P < 0.05 (**Supplementary Table 7**). Data visualization was performed using R language software (**Figure 7**). The high-risk subgroup positively correlated with tumor-infiltrating immune cells such as cancer-related fibroblasts, T cell CD8+, M2 macrophages, and macrophage and was negatively associated with T cell CD4+ and T cell follicular helper cells (**Supplementary Figure 1**).

### Correlation Analysis Between Risk Models and Immune Checkpoints

**Figure 8:**
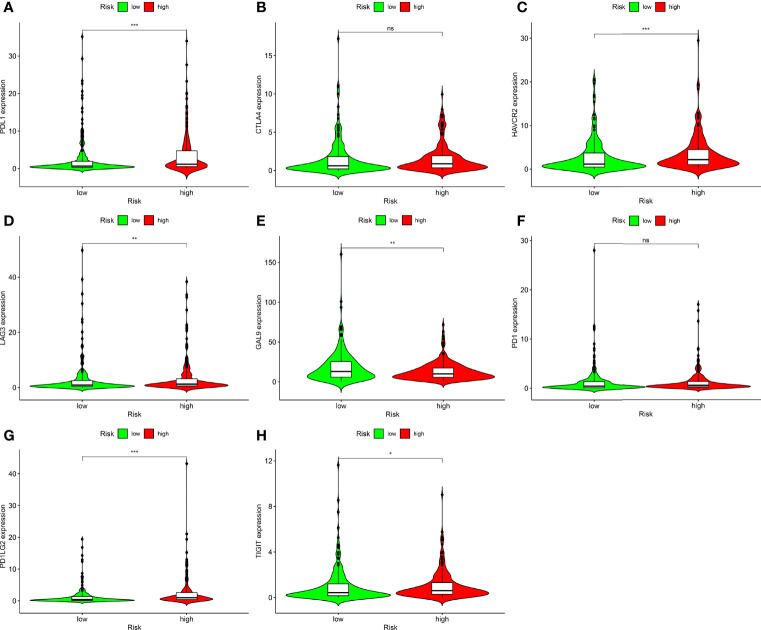
Correlation analysis between risk models and immune checkpoints of BLCA. **(A)** PDL1. **(B)** CTLA4. **(C)** HAVCR2. **(D)** LAG3. **(E)** GAL9. **(F)** PD1. **(G)** PDCD1LG2. **(H)** TIGIT in high- and low-risk BLCA patients. Ns, not significant; *p < 0.05; **p < 0.01; ***p < 0.001.

ICIs are therapeutic agents for management of BLCA. We determined whether the risk model was associated with biomarkers related to ICIs and found that PDL1 (p < 0.001; **Figure 8A**), HAVCR2 (p < 0.001; **Figure 8C**), LAG3 (p < 0.001; **Figure 8D**), PDCD1LG2 (p < 0.001; **Figure 8G**), and TIGIT (p < 0.001; **Figure 8H**) expression levels were significantly elevated in high-risk patients. Expression levels of CTLA4 (p > 0.05; **Figure 8B**) and PD1 (p > 0.05; **Figure 8F**) were increased; however, the differences were not significant. GAL9 (p < 0.001; **Figure 8E**) expression was attenuated in high-risk patients. These genes could be used as therapeutic targets for BLCA.

### Correlation Analysis Between Risk Models and Chemotherapy Drugs

**Figure 9:**
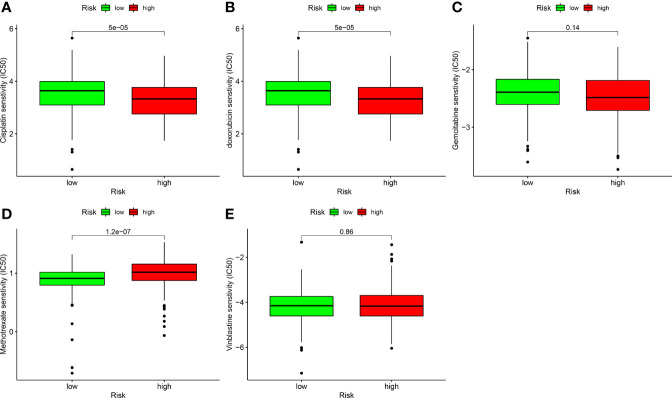
Correlation analysis between risk models and chemotherapy drugs for BLCA. Comparing the IC_50_ values of **(A)** cisplatin, **(B)** doxorubicin, **(C)** gemcitabine, **(D)** methotrexate, and **(E)** vinblastine in high- and low-risk BLCA patients.

In addition to ICIs, chemotherapy is the first-line treatment for individuals with advanced BLCA. We also explored the correlation between risk models and the efficacy of conventional cancer medicines in BLCA. We found that a higher risk score was related to a reduced IC_50_ for chemotherapy drugs including cisplatin (p < 0.001; **Figure 9A**) and doxorubicin (p < 0.001; **Figure 9B**), while it was associated with a higher IC_50_ for methotrexate (p < 0.01; **Figure 9D**), gemcitabine (**Figure 9C**), and vinblastine (**Figure 9E**). Values were not significantly different between high- and low-risk groups, suggesting that the risk model predicts chemosensitivity.

## Discussion

An imbalance between tumor cell death and growth causes tumor formation and progression (34). Excessive cell growth or prevention of natural cell death exacerbates cancer progression. Some investigators argue that immortal cell proliferation and cell death suppression are distinct characteristics of malignant tumors (35). Necroptosis is a recently discovered type of cell death with morphological characteristics similar to necrosis. By contrast, necrosis refers to passive death induced by external physicochemical stress (e.g., inflammation or infection) and is not modulated by signaling pathways, while necroptosis is governed by programmed cell death (36). Many studies linked necroptosis to cancer incidence, progression, and metastasis (37, 38). Necroptosis is also a viable strategy for eliminating cancer cells (39).

Many studies investigated the role of lncRNAs in tumor onset and progression. Abnormally expressed lncRNAs in malignancies can be used as markers for clinical diagnosis, predicting outcomes, and developing targeted therapies (34). In BLCA, lncRNAs have been linked to cancer immunology and the tumor microenvironment (35). The lncRNA urothelial carcinoma-associated 1, the most studied lncRNA in BLCA, participates in several processes in the development of BLCA and is responsible for BLCA resistance (36, 37). Based on the literature, several immune-related lncRNA models have been developed (24, 38). These signature models were created using quantification of necroptosis-related lncRNA expression. To the best of our knowledge, there are no studies exploring the relationship between necroptosis-related lncRNAs and BLCA outcomes and underlying molecular mechanisms. We identified nrlncRNA pairs and developed a robust and independent risk profile of nrlncRNAs to determine the relationship between the model and BLCA outcomes and the potential impact on the BLCA tumor microenvironment and its corresponding treatment responses.

Zhang et al. evaluated the expression levels of ten hypoxia-related lncRNAs to establish a signature predicting survival in BLCA (39). However, there is currently no study of nrlncRNAs in BLCA. Because of the critical role of nrlncRNAs, we developed a risk model with 13 DEnrlncRNAs pairs. This novel model is clinically useful, can distinguish high- or low-risk cases, and determine outcomes. In addition, we determined the correlation between the risk factor score and various clinical markers for each BLCA sample and found that the risk factor score independently predicted outcomes. We constructed ROC curves for clinically relevant indicators and compared the 1-year ROC curves in the same chart. We confirmed that the risk factor score was the best predictor of BLCA outcome at 1 year, suggesting the robustness of the risk model. Then, we evaluated each of the DEnrlncRNAs identified in our model and found that they participate in the malignant phenotype of different cancer types, while lncSNHG14 overexpression promoted breast cancer proliferation and accelerated cell cycle progression (40). Wu et al. found that lncRNA GHRLOS can be a biomarker for colorectal cancer metastasis and outcomes (41). LncRNA AC092279.1 was reported in a thyroid cancer prognostic model (42). LncRNA AC008115.3 was reported in a head and neck squamous cell carcinoma prognostic model (43). LncRNA AC010186.3 was reported in an ovarian Cancer prediction signature (44). LncRNA AC011503.2 and AC007128.1 were reported once in a bladder cancer predictive signature (45, 46). Regarding AL136531.1, AC024361.1, AL731567.1, AC018521.6, AC024361.1, and GAS6-DT, there are no reports yet.

Immune checkpoints and immune cell infiltration in cancerous tissues are critical for enhancing or inhibiting cell growth, invasion, and migration; immunotherapy is a novel therapeutic approach for managing diseases like BLCA (29). To explore the relationship between nrlncRNA signatures and immune cell infiltration, we used XCELL, TIMER, QUANTISEQ, EPIC MCPCOUNTER, CIBERSORT, and CIBERSORT-ABS algorithms to compare the content of immune cells in different risk score groups and found that the high-risk group was positively correlated with tumor-infiltrating immune cells such as cancer-associated fibroblast, T cell CD8+, M2 macrophages, and macrophages. It was negatively associated with T cell CD4+ and T cell follicular helper. Jóźwicki reported that breast cancer patients with reduced CD4+ T cell infiltration had shorter overall survival, and CD4+ T cells are a critical prognostic indicator, consistent with our findings (47). These findings suggest that this risk model could be used to anticipate the response to immunotherapy in patients with BLCA.

We also performed a correlation analysis of immune checkpoint genes and risk models and found that the PDL1, HAVCR2, LAG3, PDCD1LG2, and TIGIT expression levels were substantially higher in high-risk patients, and expression levels of GAL9 lower decreased in high-risk patients. These genes could be used as therapeutic targets for BLCA.

Bladder cancer is a complex malignant tumor, and chemotherapy is one of its essential treatment options. The guidelines recommend neoadjuvant chemotherapy before radical cystectomy of patients with BLCA, and the survival benefit of patients is close to 5–10%; nevertheless, some patients still do not respond to chemotherapy (48). Therefore, identifying predictors can avoid missing the optimal timing of surgery and minimize the adverse effects of chemotherapy. Here, we correlated BLCA chemotherapeutic agents with risk models and found that high-risk patients were more responsive to cisplatin and doxorubicin than low-risk individuals. Conversely, low-risk subjects were more responsive to methotrexate than high-risk subjects. These findings suggest that the risk model may help predict the sensitivity to doxorubicin, methotrexate, and cisplatin in patients with BLCA.

Although we employed rigorous approaches and algorithms to construct the model, this study has a few limitations. This study lacks external data to confirm the robustness of our risk model because the existing public databases do not include valid external data for verification. In a subsequent study, we will gather more clinical data and expand the sample size.

Finally, our findings revealed that a new signature created by nrlncRNAs might predict outcomes of BLCA and describe the immune landscape and chemotherapeutic therapy.

## Data Availability Statement

The datasets presented in this study can be found in online repositories. The names of the repository/repositories and accession number(s) can be found in the article/**Supplementary Material**.

## Author Contributions

The manuscript written was completed by JH, ZL, RD and GW, Experiment performance was done by GQ and YX, Data collection was conducted by HN, GY and CT, All the authors reviewed the manuscript and discussed the results and edited the manuscript. All authors contributed to the article and approved the submitted version.

## Funding

The Hunan Natural Science Foundation (#2021JJ50069) provided funding and grant its approval for the present research.

## Conflict of Interest

The authors declare that the research was conducted in the absence of any commercial or financial relationships that could be construed as a potential conflict of interest.

## Publisher’s Note

All claims expressed in this article are solely those of the authors and do not necessarily represent those of their affiliated organizations, or those of the publisher, the editors and the reviewers. Any product that may be evaluated in this article, or claim that may be made by its manufacturer, is not guaranteed or endorsed by the publisher.

## Supplementary Material

Supplementary Table 1 The clinical features of BLCA patients.

Supplementary Table 2 A list of necroptosis-related genes.

Supplementary Table 3 A list of necroptosis-related lncRNAs in BLCA.

Supplementary Table 4 The sum of 89 differentially necroptosis-related lncRNAs in BLCA.

Supplementary Table 5 3101 effective necroptosis lncRNA pairs.

Supplementary Table 6 Univariate Cox regression analysis of necroptosis-related lncRNA pairs significantly influencing outcomes in BLCA.

Supplementary Table 7 The association between risk score and tumor-infiltrating immune cells.

Supplementary Figure 1 Infiltration of immune cells in high- and low-risk BLCA samples.

## Abbreviations

BLCA, bladder urothelial carcinoma; TCGA, The Cancer Genome Atlas; nrlncRNA, necroptosis-related long noncoding RNA; DEnrlncRNA, differentially expressed necroptosis-related long noncoding RNA; AIC, Akaike Information Criterion; ICIs, immune checkpoint inhibitors; FDR, false discovery rate; LASSO, Least Absolute Shrinkage and Selector Operation; ROC, receiver operating characteristic; AUC, area under the curve; IC50, half inhibitory concentration.
