# Supplementary figures and images for: A Necroptosis-Related lncRNA to Develop a Signature to Predict the Outcome, Immune Landscape, and Chemotherapeutic Responses in Bladder Urothelial Carcinoma
Source: Front Oncol. 2022 Jun 24;12:928204. doi: 10.3389/fonc.2022.928204 (PMC9270023; doi:10.3389/fonc.2022.928204)

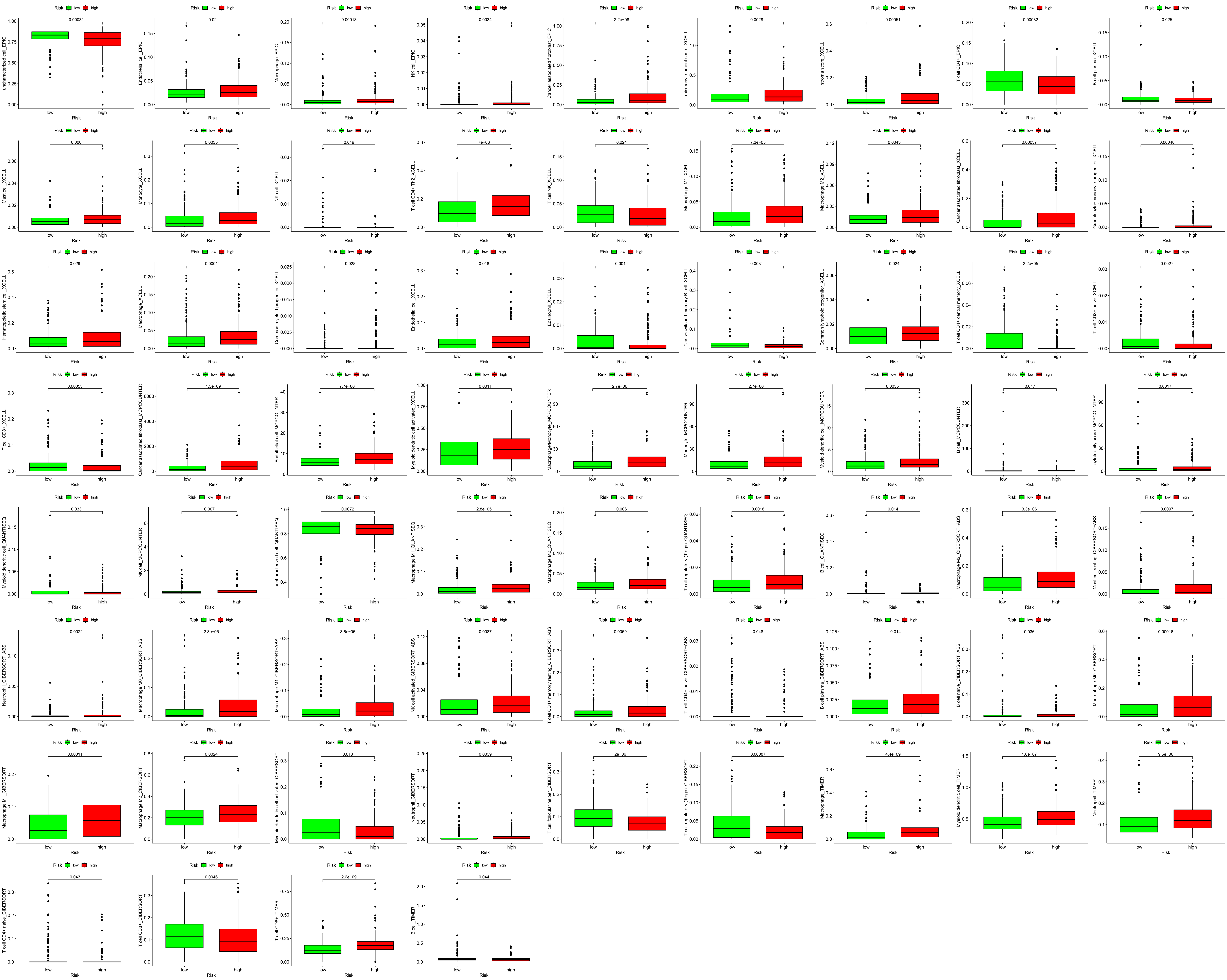

Supplement: Supplementary Figure 1 — Infiltration of immune cells in high- and low-risk BLCA samples. [file Image_1.tif]
